# Interventions to Improve Safer Sleep Practices in Families With Children Considered to Be at Increased Risk for Sudden Unexpected Death in Infancy: A Systematic Review

**DOI:** 10.3389/fped.2021.778186

**Published:** 2022-01-03

**Authors:** Catherine Ellis, Anna Pease, Joanna Garstang, Debbie Watson, Peter S. Blair, Peter J. Fleming

**Affiliations:** ^1^Department of Nursing, Midwifery and Health, Faculty of Health and Life Sciences, Northumbria University, Newcastle upon Tyne, United Kingdom; ^2^Centre for Academic Child Health, Bristol Medical School, University of Bristol, Bristol, United Kingdom; ^3^Birmingham Community Healthcare NHS Foundation Trust, Birmingham, United Kingdom; ^4^Children and Families Research Centre, School for Policy Studies, University of Bristol, Bristol, United Kingdom

**Keywords:** sudden unexpected death in infants, sudden infant death syndrome, interventions, infant safe sleep, systematic review

## Abstract

**Background:** Advice to families to follow infant care practices known to reduce the risks of Sudden Unexpected Death in Infancy (SUDI) has led to a reduction in deaths across the world. This reduction has slowed in the last decade with most deaths now occurring in families experiencing social and economic deprivation. A systematic review of the literature was commissioned by the National Child Safeguarding Practice Review Panel in England. The review covered three areas: interventions to improve engagement with support services, parental decision-making for the infant sleep environment, and interventions to improve safer sleep practices in families with infants considered to be at risk of SUDI.

**Aim:** To describe the safer sleep interventions tested with families with infants at risk of SUDI and investigate what this literature can tell us about what works to reduce risk and embed safer sleep practices in this group.

**Methods:** Eight online databases were systematically searched in December 2019. Intervention studies that targeted families with infants (0–1 year) at increased risk of SUDI were included. Studies were limited to those from Western Europe, North America or Australasia, published in the last 15 years. The Quality Assessment Tool for Studies with Diverse Designs was applied to assess quality. Data from included studies were extracted for narrative synthesis, including mode of delivery using Michie et al.'s Mode of Delivery Taxonomy.

**Results:** The wider review returned 3,367 papers, with 23 intervention papers. Five types of intervention were identified: (1) infant sleep space and safer sleep education programs, (2) intensive or targeted home visiting services, (3) peer educators/ambassadors, (4) health education/raising awareness interventions, (5) targeted health education messages using digital media.

**Conclusion:** Influencing behavior in families with infants at risk of SUDI has traditionally focused on “getting messages across,” with interventions predominantly using education and awareness raising mechanisms. This review found evidence of interventions moving from “information giving” to “information exchange” models using personalized, longer term relationship-building models. This shift may represent an improvement in how safer sleep advice is implemented in families with infants at risk, but more robust evidence of effectiveness is required.

**Systematic Review Registration:** https://assets.publishing.service.gov.uk/government/uploads/system/uploads/attachment_data/file/901091/DfE_Death_in_infancy_review.pdf, identifier: CRD42020165302.

## Introduction

A baby dying suddenly is devastating for any family. The ramifications spread to wider friends and family, and to health care professionals who supported them during the first months of the baby's life (1). Sudden unexpected death of an infant (SUDI) is the term used at the point of presentation and includes deaths for which a cause will be identified, such as infection, and those that cannot be fully explained and are categorized as sudden infant death syndrome (SIDS) (2) or unascertained, accounting for ~200 infant deaths annually in England and Wales (3). The demographic profile of these deaths now reveals an inequity gradient, with younger parents living in socio-economic deprivation experiencing the highest rate of infant deaths at 1.18 per 1,000 live births, more than four times the rate in the general population (3). Several characteristics have been associated with higher rates of SUDI which include vulnerable infants (low birthweight, pre-term, multiple births, and admission to NICU), young maternal age, smoking exposure during and after pregnancy, bottle feeding, male preponderance, and lower socio-economic status (3–5). The peak age of death is not the first few weeks of life when infants are at their most vulnerable but at 2–3 months of age. Observational evidence over the last 30 years has identified risk factors pertaining to the infant sleep environment that, when modified, have been shown to reduce the risk of some infant deaths (6). These risks include placing infants to sleep on their side or front, using too many and/ or loose bedclothes, solitary sleep room in the first 6 months, and specific hazardous circumstances for bed-sharing and co-sleeping, such as infants sleeping next to carers who smoke, have consumed alcohol or drugs, or share inappropriate surfaces, for example, sofas; or bedsharing or co-sleeping with a baby born with a low birthweight or pre-term (4, 6, 7).

Some of the background characteristics and recognized risks for SUDI overlap with, but are not predicted by, those of child maltreatment, and families with children who may be at risk of abuse or neglect often face multiple vulnerabilities, including risks of SUDI (8). A recent thematic analysis of 27 SUDI cases leading to Serious Case Reviews in England (9), found families had complex social backgrounds, with long-term neglect, alcohol or drug misuse and non-engagement with services as a prominent feature. The review also identified that safer sleep advice was only documented in half of these families. One of the key challenges in working with high-risk families is not limited to just sharing safer sleep advice, but ensuring the evidence underpinning these messages is better communicated to, and understood by parents, and implemented into both usual and out of routine parenting practices. Out of routine situations which change the infant sleep environment can unintentionally increase risk for infants where make-shift sleeping arrangements or co-sleeping may be the only option and particularly where the priority is to achieve sleep for both infant and parent rather than consider the safety of the sleep environment (10–15). Understanding how best to reach and engage vulnerable families to adopt safer infant care practices has been highlighted in previous research (14–16), however, identifying the most effective interventions or methods to achieve this, or identifying the effective components of interventions that are successful are lacking (17). The second National Child Safeguarding Practice Review (NCSPR) (18) focused on the occurrence of SUDI in families where children were considered to be at risk of abuse or neglect, aiming to identify the most effective methods for professionals to provide effective support to ensure that safer sleep advice can be clearly understood and embedded. As part of their work, the NCSPR Panel commissioned a systematic review in three key areas (19): (1) interventions to improve engagement with support services (20), (2) improving our understanding of parental decision-making processes related to the infant sleep environment (21), and (3) the evidence on interventions for improving the uptake of safer sleep advice, which is the subject of this paper.

This systematic review focuses on the third key area addressing the research question: what safer sleep interventions have been tested for families with infants at risk of SUDI, and what can these tell us about what works to reduce the risk and embed safer sleep practices for infants at higher risk?

## Methods

The review protocol was registered with the International prospective register of systematic reviews, PROSPERO number: CRD42020165302. We focused our review on families with children considered to be at high risk for SUDI, which may significantly overlap with the wider group of families with children considered to be at high risk of significant harm through abuse or neglect. The population of interest included families with infants under the age of 1 year and considered to be at high risk of SUDI, however defined by individual studies. Inclusion criteria for what constituted “high risk” populations were wide due to the variability of definitions within individual studies. We included all studies that took a targeted approach to intervention and included interventions aimed at improving infant safer sleep practices and included those which sought to influence the infant sleep environment, rather than those aimed at reducing risks such as stopping smoking or increasing breastfeeding. We therefore included interventions with an aim to have any impact on infant sleep position, co-sleeping, bed-sharing, dummy/pacifier use, swaddling, room sharing, infant bedding, exposure to tobacco smoke in the home, or room temperature. Where studies tested an intervention, the comparator was expected to be either standard care or a less intensive version of the intervention.

Our search strategy included terms relating to our population, outcome of interest and intervention terms. Our sample search terms are shown in Appendix 1. Our inclusion criteria at screening limited studies of interventions to those reported in the last 15 years and those form Western Europe, North America or Australasia. Given that infant care practices change over time, a scope of 15 years was felt to be reasonable to capture the current practices of parents and carers. One of the main aims of the review was to describe the literature on interventions relevant to the UK population, which meant that consideration for the context in which interventions took place was a relevant factor. While we did not wish to ignore effective interventions from other parts of the world, we did want to focus on those which had been developed and evaluated within broadly similar cultural contexts and infant care practices.

Unpublished reports were included where they met the inclusion criteria and included data on the results or outcomes of the study. Other exclusion criteria included papers relating to explained non-sleep causes of death, for example infections or metabolic disorders found at post-mortem (non-relevant outcome); studies describing interventions for the general population with no high-risk targeting (non-relevant population) and studies describing interventions not related to safer sleep or the sleep environment (non-relevant intervention).

The review was conducted in December 2019 and eight online databases were searched (see Appendix 1). Additional searches for gray literature and relevant interventions were conducted in January 2020, by emailing all English Child Death Overview Panels, Designated Doctors for Child Death and Safeguarding, UK safeguarding children's partnerships, and the membership of The International Society for the Study and Prevention of Perinatal and Infant Death, a global non-profit organization of researchers, health professionals and parents. Further snowball searches of included and relevant papers' reference lists were also conducted.

Four authors (AP, JG, CE, DW) scoped the initial search terms and refined a final list of terms for inclusion in each search by assessing the first 30 titles and abstracts in Medline for relevance and other terms. Titles and abstracts were deduplicated in Endnote and imported into Rayyan, online screening software (https://rayyan.qcri.org/). All returned titles and abstracts were screened by four authors (AP, JG, CE, DW), applying the inclusion and exclusion criteria, and conflicts were resolved by examination of the full text and discussion. All included texts were sourced, and the quality of papers assessed using the Quality Assessment Tool for Studies with Diverse Designs (QATSDD) (22). This approach was developed specifically for review questions where the evidence addressing a research question uses a variety of different study designs. The tool is used across both quantitative and qualitative research designs, to facilitate assessment of the quality of studies comparatively across all included studies. Four team members (AP, JG, CE, DW) scored each paper from 0 to 3 on either 14 or 16 items (depending on study design) and converted each score into a percentage. Included papers of review author's own work were independently rated by another team member. Given the expected paucity of data in this field studies were not excluded based on quality assessment but limitations to the findings are discussed where necessary.

Data extraction templates were piloted and refined for use with nine of the included papers of different study designs. The final data extraction form included fields for author's names, year of publication, study design, country, sample size, target population, type of outcome, comparator, outcomes measured and effectiveness. Specific fields for qualitative studies included method of analysis and broad topic categories. For the intervention papers, the mode of delivery was extracted using variables influenced by Michie et al.'s Mode of Delivery Taxonomy (23) and collected data on whether interventions were face to face, on printed material, digital, used equipment, delivered individually, in groups, involved one-way or two-way interaction, and whether they were tailored, meaning that the intervention was responsive to, or changed depending on circumstances of participants. Popay et al.'s (24) framework for conducting narrative reviews is used to standardize narrative approaches to systematic reviews, where the primary synthesis comes from understanding how and why an intervention worked or did not work, rather than meta-analysis which was not possible in this review given the heterogeneity of the reported results. Narrative synthesis offers a systematic approach to evaluating both outcomes and processes in intervention studies and is therefore particularly relevant in the review of these papers.

## Results

**Figure 1:**
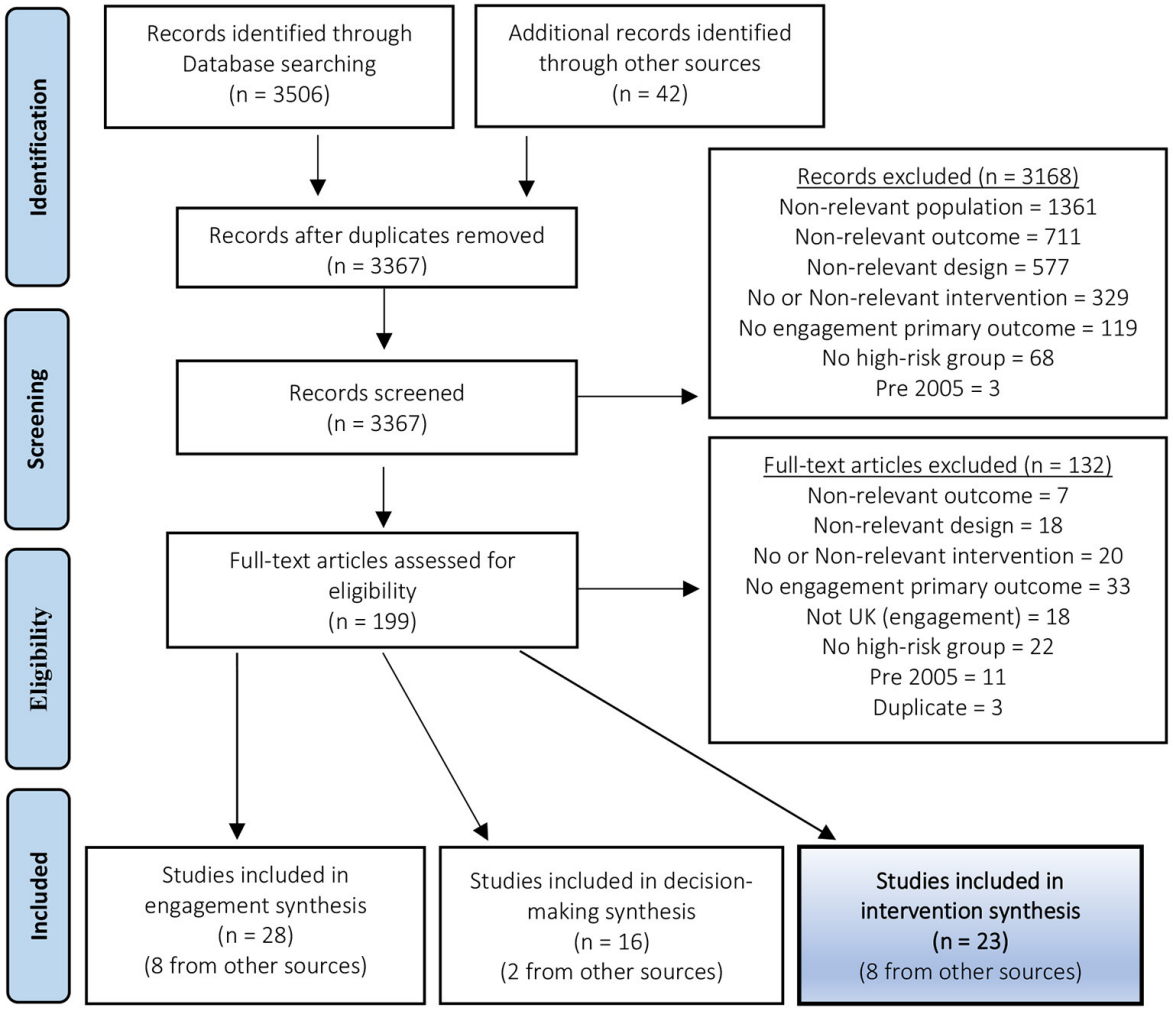
PRISMA flow diagram of literature search and selection process.

Following de-duplication in Endnote, a total of 3,367 records were screened. Ten percent of records (324 records) were screened by two authors with a 97% agreement rate. Twenty-four conflicts were resolved through discussion and examination of the full text. Duplicates identified at the full text screening stage were conference abstracts from studies that were included as full text papers. Sixty-seven papers were included in the systematic review, 23 of which identified interventions to reduce the risk of SUDI in high-risk families (Figure 1).

Twenty-three papers of interventions with populations identified as vulnerable were included for synthesis and are grouped by intervention type in Table 1.

**Table 1:** Characteristics of included interventions to reduce the risk of SUDI in families with children considered to be at high risk.

**References Country**	**Study design and sample size**	**Target population**	**Intervention/ control**	**Study aim**	**Mode of delivery**	**Key findings/ measure of success**	**QATSDD score/%**
**Infant sleep space and education program**							
Baddock et al. (25) New Zealand	RCT 98 I/V 101 Control	Maori pregnant women living in low socio- economic areas	Provision of a woven flax bassinet (Wahakura) designed to provide a consistent infant sleep environment. Control: Usual bassinette	To compare an indigenous sleep device (Wahakura) for infants at high risk for sudden unexpected death with a bassinet, for measures of infant sleep position, head covering, breastfeeding, bed- sharing, and maternal sleep and fatigue.	Face to Face Printed material Infant sleep space Individual	No significant differences in infant risk behaviors in Wahakura compared with bassinets. Increase in sustained breastfeeding in the Wahakura group.	29/42 69.0%
Carlins et al. (26) USA	Evaluation 150	Low-income families	Crib distribution and safe sleep education	Evaluate Cribs for Kids campaign; crib distribution and safe sleep education	Face to Face Printed material Infant sleep space Individual	100% reported use of the distributed crib. No SUDI deaths reported for the crib distribution families (still resident in locality).	18/42 42.9%
Young et al. (27) Australia	Test of concept trial 158	Aboriginal and Torres Strait Islanders	Pepi-Pod program	Pepi-Pod program evaluation	Face to Face Printed material Infant sleep space Individual	Pepi-Pods acceptable to and used by families; improved safe sleep recommendation adherence.	25/48 52.1%
Engel et al. (28) USA	Pre-post surveys and observation 75	Need was determined holistically by maternal infant health program (MIHP) staff, with indicators including low- income, racial minorities, and migrant worker status.	Crib distribution and safe sleep education	Identify changes in knowledge and how many parents used the cribs provided by Crib distribution program	Face to Face Printed material Infant sleep space Individual	99% using the distributed crib. Increased knowledge supine position (59% pre−89% post).	27/42 64.3%
Cowan (29) New Zealand	Evaluation of program implementation 3,616	Infants aged < 2 weeks, smoke- exposed, premature or low birth weight, with local discretion for exceptions based on safety assessments of the care-giving professional	Pepi-Pod program	To examine distribution, follow-up and user- feedback records	Face to Face Printed material Infant sleep space Individual	Maori IMR decreased. Pepi-Pods acceptable to and used by families; improved safe sleep recommendation adherence.	31/48 64.6%
Hauck et al. (30) USA	Prospective cohort study 6,515	(1) no crib in the home; (2) low income status (3) at least one risk factor for SIDS and sleep-related death (ethnicity, maternal smoking, pre- term or low birth weight, or sibling of a SIDS infant)	Crib distribution and safe sleep education	Evaluate Bedtime Basics for Babies campaign; crib distribution and safe sleep education	Face to Face Printed material Infant sleep space Individual	Knowledge of sleep position improved from 76 to 94%, bed-sharing decreased from 38 to 16%, 90% of parents used a crib.	32/42 76.2%
Yuill et al. (31) UK	Feasibility study 79 I/V 70 Control	Young parents, parents who had smoked in pregnancy, and those known to be substance users.	Plastic baby box bed and safe sleep education Control: Usual care	Feasibility study for RCT to introduce UK version of Pepi-Pod program	Face to Face Printed material Infant sleep space Individual	Intervention reduced sofa co-sleeping to 6 vs. 23% of controls and decreased mean bed-sharing hours to 2.6 per night compared to 6.8 for controls.	21/48 50.0%
McIntosh et al. (32) New Zealand	RCT 101 I/V 110 Control	Maori and Pacific women	Pepi-Pod program Control: Better than usual care and infant sleep space	Assess acceptability and effectiveness at improving SUDI protective knowledge and safe sleep practice from the Pepi- Pod program compared to usual care	Face to Face Printed material Infant sleep space	Improvements seen in both I/V and control groups due to more than usual care provision for control group, as all participants were provided a cot.	25/42 59.5%
Salm Ward et al. (33) USA	Cohort study 208	High-risk parents (demonstrated financial need)	Crib distribution and safe sleep education	Compare parental knowledge and practices related to infant sleep before and after receipt of safe sleep educational programme and receipt of a crib	Face to Face Printed material Infant sleep space Group Interactive	Knowledge of recommendations on position, surface, environment, pacifier, smoking and breastfeeding increased significantly between pre and post-test and most maintained knowledge at follow-up.	24/42 57.1%
**Intensive home visiting or targeted services**							
Dillon (34) UK	Service Case Study 1,047	Alcohol/ substance misuse, violent criminal history, previous child not living with parent, late ante natal booking, homelessness with mental health/domestic abuse/probation, hearing impaired.	Vulnerable baby service: multi agency case planning meetings, and a public health approach.	Engage vulnerable families in the design of their support package with the aim to reduce risks of SUDI	Face to Face Interactive	Infant deaths reduced by 60% in Manchester. SUDI rate decreased from 1.8/1,000 to 0.52 in 2011.	10/42 23.8%
Hutton et al. (35) USA	RCT 160 I/V 122 control	Low SES mothers	Home visiting education with Baby Book Control: Usual brochures for safe sleep knowledge	To test the efficacy of a specially designed children's book compared to brochures for safe sleep knowledge and adherence	Face to Face Printed material Individual	Safe sleep knowledge increased cross all time points for both groups. Bed-sharing was higher and exclusive crib use lower in the brochure group. Greater dialogue and emotional engagement were reported with use of the book.	30/42 71.4%
Kemp et al. (36) Australia	RCT 111 I/V 97 Control	Vulnerable parents: one of a list of risk factors	Maternal Early Childhood Sustained Home- visiting (MECSH) Program Control: Usual care	To develop a theory of change for pre-natal home visiting by nurses in the context of sustained nurse home visiting	Face to Face Individual Interactive	Less instrumental deliveries; improved health and well-being scores; improved coping and self-efficacy in parenting in the	30/42 71.4%
				programs by exploring pre- and postnatal outcomes and the characteristics of the MECSH program intervention		intervention group.	
Olds et al. (37) USA	RCT 458 I/V 680 Control	African American mothers living in highly disadvantaged urban neighborhoods	Nurse-Family Partnership Control: Usual care	All-cause maternal mortality and preventable- cause infant mortality	Face to Face Individual Interactive	Intervention group mothers less likely to die from all-causes and offspring less likely to die from preventable causes.	35/42 83.3%
**Peer educators**							
Cowan (38) New Zealand	Evaluation of a pilot study 7	Women and their partners who had successfully quit smoking during pregnancy	6 + 1 peer education	To achieve high levels of awareness of 6 + 1 information in communities that make low use of traditional health services, to achieve 50 “6 + 1” conversations in 1 month	Face to Face Printed materials Individual Interactive	Link workers (parents) reported 70 6 + 1 conversations; total of 90 6 + 1 conversations reported at evaluation. Hard to reach became “easy to reach” by changing the communication paradigm.	28/48 58.3%
Gilchrist (39) UK	Evaluation of web-based peer support for young parents 55	Young parents	Little Lullaby project: raise awareness and reduce risk for SIDS in young parents	Young parents adopt and feel confident in applying the Lullaby Trust's recommended “safer sleep for babies” advice	Face to Face Digital	97.5% of young parents learned about safe sleep and SIDS risk reduction; some parents changed behavior as a result.	30/48 62.5%
**Health education interventions**							
Ahlers-Schmidt et al. (40) USA	Evaluation surveys 180	African American women	Safe sleep community baby shower	To describe participants' knowledge and intentions regarding safe sleep following a Community Baby Shower	Face to Face Printed material Infant sleep space Group	High levels of safe sleep knowledge and stated intentions to follow safe sleep recommendations were reported by participants.	27/42 64.3%
Ahlers-Schmidt et al. (41) USA	Evaluation surveys 845	Pregnant women of low socioeconomic status or with high risk of infant mortality	Safe sleep community baby shower	To evaluate outcomes of Safe Sleep Instructor-led community baby showers, which included safe sleep promotion, breastfeeding promotion and tobacco cessation education.	Face to Face Printed material Infant sleep space Group	Significant increases were observed in Baby Shower participants' reported plans to follow the AAP Safe Sleep guidelines (all *p* < 0.001).	26/42 61.9%
Ostfeld et al. (42) USA	Pre-post intervention surveys 810	Adolescents/ parents	High school education program	Improve SIDS risk knowledge	Face to Face Group	Awareness that supine sleep position carried less risk and infant smoke exposure increased risk of SIDS improved post intervention.	14/42 33.3%
Burd et al. (43) USA	Pre-post intervention surveys 341	Native American women	Discussion covering 9 risk factors, provision of a printed baby blanket and printed materials.	To complete a community-based efficacy study of a SIDS risk reduction methodology.	Face to Face Printed material Individual	Pre-test identified significant safe sleep knowledge deficit, higher in Native American group. Intervention improved knowledge on all nine items in both groups	24/42 57.1%
Rienks et al. (44) USA	Telephone surveys following campaigns 1,458	African Americans 18–64 yrs	3 media campaigns	Evaluate campaign effectiveness in African Americans	Digital Leaflet Posters	Exposure to 3 campaigns was successful in raising awareness of IM disparity in African Americans.	32/42 76.2%
**Targeted education messages** ***via*** **digital media**							
Carlin et al. (45) USA	RCT 569 I/V 625 Control	African American mothers	Targeted and enhanced safe sleep messages Control: Standard messaging emphasizing AAP recommended safe sleep practices	Evaluate the impact of targeted messages about safe sleep and SIDS risk reduction on African American mothers decisions regarding the infant sleep environment: Sleep position	Digital	Supine position use decreased over time. Behavior unchanged by enhanced message intervention.	30/42 71.4%
Mathews et al. (46) USA	RCT 569 I/V 625 Control	African American mothers	Targeted and enhanced safe sleep messages Control: Standard messaging emphasizing AAP recommended safe sleep practices	Evaluate the impact of targeted messages about safe sleep and SIDS risk reduction on African American mothers decisions regarding the infant sleep environment: Soft bedding	Digital	Decrease in use of soft bedding in the intervention group: previous night 43.0 vs. 52.4% in controls and over previous week 49.2 vs. 59.6% in controls.	26/42 61.9%
Moon et al. (47) USA	RCT 569 I/V 625 Control	African American mothers	Targeted and enhanced safe sleep messages Control: Standard messaging emphasizing AAP recommended safe sleep practices	Evaluate the impact of targeted messages about safe sleep and SIDS risk reduction on African American mothers decisions regarding the infant sleep environment: Sleep location	Digital	Women receiving enhanced messages were no less likely to bedshare: no effect of intervention.	25/42 59.2%

From these 23 publications, over half of the studies (14/23) were conducted in the USA (26, 28, 30, 33, 35, 37, 40–47), four in New Zealand (25, 29, 32, 38), three in the UK (31, 34, 39), and two in Australia (27, 36). The studies span 14 years from 2005 to 2019 and the overall quality scores ranged from 23 to 83%, with 20/23 papers scoring 50% and above. The paper scoring 23.8% was a short descriptive digest, a “case study” of good practice describing the intervention and key outcomes, rather than a research paper (34). The majority of these studies were quantitative; eight were randomized controlled trials (25, 32, 35–37, 45–47) and six were evaluations (26, 29, 38–41); the remainder were mixed methods or used a variety of quantitative approaches. Three papers utilized the same research data set, but presented different outcomes (45–47). The number of participants ranged from seven (38) to 6,515 (30) and participants were pregnant women, mothers or families identified to have some vulnerability, or characteristics that increased risk of SIDS to their infants. Seven studies recruited based on ethnicity alone (27, 32, 40, 43, 45–47), with ethnicity being used as a marker for deprivation or increased risk due to socioeconomic status.

From these 23 results, five types of intervention were identified which are discussed below:

1. Infant sleep space and safer sleep education programs – 9 papers (25–33)
2. Intensive or targeted home visiting services – 4 papers (34–37)
3. Peer educators/ambassadors – 2 papers (38, 39)
4. Health Education/Raising Awareness Interventions – 5 papers (40–44)
5. Targeted health education messages using digital media – 3 papers (45–47)

### Infant Sleep Space and Safer Sleep Education Programs

Nine papers (25–33) reported on the provision of a safe infant sleep space (crib, Pepi-Pod^®^, Wahakura or plastic box baby bed) with a safer sleep educational component, aiming to improve parental safe sleep knowledge and influence behavior to reduce the risks of hazardous infant sleep environments. Studies investigated safe sleep devices for both use external to the parental bed (cribs) (26, 28, 30, 33), and devices intended as a separate safe sleep space for the infant, but for use within the parental bed (Pepi-Pod^®^, Wahakura or plastic box baby bed) (25, 27, 29, 31, 32).

There were a number of study designs within this theme comprising of mixed methods evaluations of cohort studies based on parental self-report behavior and/or intention data (26, 28, 30, 33) two RCT's (25, 32), two feasibility studies (27, 31) and one report of intervention implementation (29).

Four studies evaluated crib distribution and safer sleep education programs in the USA (26, 28, 30, 33). Carlins and Collins (26) found that all participants used the crib provided, commenting that 38% of participants at enrolment did not have a crib and would have bedshared. All participants reported attending all well baby checks however, only 65% of parents stated they placed their infant supine to sleep, and although all participants claimed to have read the educational information, 50% could not explain SIDS. Engel et al. (28) reported that 99% of participants used the crib, and knowledge of supine sleep position increased from 59 to 89% following education. Hauck et al. (30) found that knowledge of sleep position improved from 76 to 94%, bed-sharing decreased from 38 to 16%, and 90% of parents used a crib for infant sleep. Salm Ward et al. (33) found that self-reported parental knowledge on risk factors for sleep position, sleep surface, sleep environment, pacifier use, smoking and breastfeeding all increased significantly following intervention, and participants demonstrated that knowledge was retained at 10-week follow up.

Five studies investigated devices intended as a separate safe sleep space for the infant, but for use within the parental bed (25, 27, 29, 31, 32). These devices included the Wahakura, a traditionally woven flax basket baby bed (25) and the Pepi-Pod^®^, a plastic box supplied with appropriate bedding (27, 29, 31, 32). Baddock et al. (25) investigated the use and acceptability of the Wahakura compared with usual bassinette use in the control group, concluding that the Wahakura increased the safety to the infant of bed-sharing, with the advantage of increasing breastfeeding rates. Three studies (27, 29, 32) reported on the Pepi-Pod program, originating in New Zealand, which involves the provision of a safe infant sleep space (plastic box) and a SIDS risk reduction education session delivered face to face by the provider. Parents are encouraged to pass on the Pepi-Pod and share the SIDS risk reduction messages with the new owners. Pepi-Pods in some studies also had safe sleep guidance labels stuck to them to facilitate sharing of accurate safer sleep messages. Cowan (29) reported that the program was applied consistently, Pepi-Pods were accepted, used, and liked by parents and were portable. Follow up demonstrated high uptake of safer sleep (supine position and infant placed in their own sleep space) and safe baby (immunization, breastfeeding, gentle handling, being smoke-free or receiving support to quit, and registration with health services) outcomes, and 80% of recipients reported sharing safer sleep messages across their networks. McIntosh et al. (32) investigated the impact of the educational element of the program on SUDI protective knowledge and infant care practices, and the acceptability of the Pepi-Pod as an infant sleep space. One quarter of participants did not have a suitable sleep space for their infant at enrolment to the study. McIntosh reported that knowledge of smoking and bed-sharing as risks for SUDI improved post intervention in both groups, however, 25% of participants reported regular bed-sharing at follow-up in both groups. All families, both intervention and control group parents, were supplied a Pepi-Pod and safe sleep education; the control group in effect received better than usual care, therefore it was difficult to assess efficacy of this element of the program by comparison to the control group in this study. Young et al. (27) evaluated the Pepi-Pod program in Australia, reporting improvements in quality of maternal sleep; breastfeeding; convenience and ease of use, and improved infant settling. Fifty-seven percentage of smoking families reported using the Pepi-Pod. A feasibility study of introducing a similar intervention based on the Pepi-Pod program in the UK was conducted by Yuill et al. (31). They reported mixed reviews but generally, parents liked the concept, and would recommend its use. Yuill identified less exposure to some hazardous sleep environments such as sofa sharing at 1 month (6 vs. 23% control) and co-sleeping with overly tired parents at 13 vs. 27% in controls.

### Intensive or Targeted Home Visiting Services

Four studies investigated intensive or targeted multi-modal home visiting interventions (34–37); two were RCTs (35, 37); one process evaluation (36) and a short descriptive “digest” of a citywide intervention (34). These interventions shared characteristics such as incorporating evidence-based elements and frameworks for service delivery shown to reduce the impact of biological, social, and environmental factors predisposing infants and children to ill health and reducing their life potential. Due to their intensive and longitudinal nature, these interventions are based on building a relationship between professional and service recipient, and as such facilitate constructive conversations and education/ advice giving based on the needs of the family. Hutton et al. (35) tested the efficacy of a specially designed children's book compared to usual brochures (advice leaflets) for safer sleep knowledge and adherence to safer sleep practices. Home visitors provided safer sleep teaching and assessments during 3 visits. Results showed that safer sleep knowledge improved across all time points in both groups, however, exclusive crib use and reduced bed-sharing was greater in the intervention group which was attributed to the enhanced dialogue and emotional engagement with the book content, suggesting that the relationship between professional and parent was a key factor. Benefits of the book were identified as the interactive delivery, and 81% of the intervention group were reading the book with their infant at 2 months. The researchers posit that emotional engagement with the book content might support the translation of knowledge into behavior and identified the benefits of access to the home provided an ecological view of how safer sleep knowledge may be assimilated and translated into adherence. Three interventions were delivered by midwives and specialist nurses, beginning in the antenatal period, and continuing well into the postnatal period or up to 2 years (34, 36, 37). Olds et al. (37) reported on 20-year follow up data on the Nurse Family Partnership (USA). The Nurse Family partnership was launched in 1990 aiming to improve life chances and outcomes for families in the poorest communities in the USA and improve the associated mortality rates influenced by racial and economic disparity. The intervention aimed to tackle through education, issues of maternal smoking and substance use, encouraged healthy spacing of pregnancies, supported parenting capability, and facilitated young mothers into further education. Mortality rates were used as an outcome measure to assess the efficacy of the program due to higher rates of mortality being related to SIDS, unintentional injuries and homicide in children of the target population. Using maternal all-cause mortality and child preventable-cause mortality outcome measures, women in the intervention group were less likely to have died and their children were much less likely to die of preventable causes such as SIDS, unintentional injuries, and homicide however, this was a small sample from which to make inferences about mortality. The Vulnerable Baby Service (34) delivered in Manchester, a large English city, aimed to engage vulnerable families in the design of their support package with the objective to reduce risks of SUDI. Since the start of this multi-agency service in 2003, the infant death rate in Manchester, UK has declined by 60% and no SUDI have been reported in the intervention group, however, no causal association is identified in the paper. Parental attendance at appointments improved, disclosure of domestic abuse increased, and 86% of fathers continue to be involved in families. Organizational benefits of increased staff engagement to reduce SUDI, attendance at SUDI training and a consistent workforce approach to delivering safer sleep advice were also observed. Kemp et al. (36) conducted a process evaluation on a program theory for pre-natal home visiting by nurses in the context of a sustained nurse home visiting program. Kemp explored pre and postnatal outcomes and characteristics of the intervention that may have contributed to the outcomes. She found that mothers in the intervention group reported significantly better general health and well-being at 4–6 weeks post-partum, and a significantly higher proportion could identify two or more measures to reduce the risk of SIDS compared to controls. In identifying intervention characteristics, Kemp noted that comprehensive support in the context of an enabling client-nurse relationship and continuity of carer, achieved both clinical and improved service engagement benefits for women and their infants.

### Peer Educators/Ambassadors

Two papers evaluated interventions with peer educators (38, 39). An infant health promotion activity in New Zealand (38) aimed to support link workers (parents) from the community to have focused discussions, supported by a baby book resource, with family and friends on key health topics to raise awareness in communities that make low use of traditional health services. The “pay-it-forward” principle of this project aimed to create a “ripple effect” of knowledge transfer to penetrate deeper into communities by using members of that community to share health education messages; this principle was observed to create leverage in sharing health education within the community. Link worker experiences were positive, the baby book was designed as an easy read, compact and colorful prompt for conversations based on the “Facts for Life” publication by UNICEF/WHO and UNESCO (48), and covered topics including a smoke-free pregnancy and environment, back sleeping in a safe sleep space, breastfeeding and the benefits of reading to your infant. The book supported and structured conversations and was valued, and information was received well by friends and family. This intervention provides an easily scalable reach for safer sleep messages into traditionally “hard to reach” communities, however, one of the concerns with this method of intervention was the loss of control and fidelity of information being shared by link workers, and difficulties in recruiting men as link workers (38). Gilchrist (39) evaluated an intervention provided by Little Lullaby, a subsidiary of The Lullaby Trust, a UK SIDS prevention charity. Little Lullaby trains young parents as Ambassadors to deliver safer sleep advice and work with young people and professionals to raise awareness and reduce risks of SIDS. The service is delivered *via* a website and face to face talks and workshops. Evaluation of the intervention indicates that safer sleep messages are being understood and applied by young parents, with 97.5% reporting they had learnt something new about safer sleep and SIDS, and 36.7% of young parents would change their parenting practice because of the session. Benefits of the intervention include providing an effective model for engaging and empowering young parents, however, at the time of the evaluation, the Ambassador program was based in London and a survey of relevant health professionals found that awareness of this scheme and the work of Little Lullaby was reported to be relatively low.

### Health Education/Raising Awareness Interventions

All five studies in this section were conducted in the USA (40–44); two were evaluations (40, 41), two were pre and post-test designs (42, 43) and one tele-survey (44). The focus of these studies was on health education or raising awareness, and although Ahlers-Schmidt et al. (40) provided a cot to participants, this was not the focus of their study. While specific educational elements are presented here, it is acknowledged that there is some potential for overlap between these studies and those reported in theme 1. Ahlers-Schmidt et al. (40) evaluated safer sleep community “baby showers” designed to increase knowledge and practice of safer sleep advice and promote social cohesion; participants were also given portable cots. While knowledge of safe sleep and intentions for safe infant care were high, no baseline measure or use of controls means that changes in knowledge or intentions due to the intervention could not be assessed. In a later study of knowledge, confidence, and intentions to follow safer sleep recommendations, Ahlers-Schmidt et al. (41) found significant increases in participants' reported plans to follow the American Academy of Pediatrics Safer sleep guidelines however, these were again parental self-reported intentions, not a reflection of actual infant-care practice. However, 86.4% of mothers reported their infant would have slept in an alternative potentially hazardous sleep space, had they not received the cribs. Burd et al. (43) evaluated an educational intervention delivered by hospital nurses or home visiting staff, where nine SIDS risk factors were discussed. Many participants had young children, therefore there was expectation that parents already had some knowledge regarding recommended safer sleep practices, however at base-line testing, substantial knowledge deficits were identified in both groups. Following intervention, participants from both groups demonstrated equivalent rates of learning across each of the risk concepts. An evaluation by Ostfeld et al. (42), of an interactive high school program to address health risks associated with smoke exposure and non-supine infant sleep, found that students were able to recognize specific risks for SUDI, retained that knowledge over time, and demonstrated better knowledge of SUDI risk factors than a convenience sample of first-time parents. Reinks and Oliva (44) evaluated three multi-media campaigns to raise awareness of infant mortality disparity in black infants. Reinks concluded that social marketing is an effective tool to increase disparity awareness, especially among groups disproportionately affected by the disparity, however, no overall significant increase in knowledge about sleep position was identified.

### Targeted Health Education Messages Using Digital Media

Three papers (45–47) reported on different aspects of the results from a RCT which evaluated the impact of targeted safe sleep messages in the USA (45). Controls were sent standard text messages emphasizing recommended sleep practices while the intervention group received enhanced messages to include suffocation prevention. Results identified a decrease in use of supine sleep position (45) and a gradual increase in bed-sharing (47) over time and in both groups, despite families being in trial conditions advising the opposite, and despite reported good parental knowledge of the recommended sleep position. Commonly cited reasons for using sleep positions other than the recommended supine position were fear of suffocation, choking and infant preference. Some influence was noted on maternal selection of supine sleep position if nurses had discussed sleep position with the mothers, however, where mothers discussed this with the father of their infant, these mothers were more likely to select prone position and over time, the opinion of maternal friends became more significant on influencing choice of sleep position. Matthews et al. (46) found a decrease in the use of soft bedding where mothers “believed” that soft bedding increased the risk of suffocation or SIDS, while mothers who were more likely to use soft bedding, including mothers who bed-shared, cited “vigilance” as protective.

The main findings presented here suggest that the most convincing evidence for interventions that work have a number of identifiable characteristics which are: personalized, culturally sensitive, enabling, empowering, relationship building, interactive, accepting of parental perspective, non-judgmental and are delivered over time (Table 2).

**Table 2:** Intervention characteristics matrix.

	**Sleep device**	**Education**	**SS knowledge improvement**	**Behavior change element**	**Home visits**	**Inter active**	**Parent perspective**	**Empowering**	**Digital**	**Peer educator**	**Group**	**Intervention reported successful**
Gilchrist (39)		•	•	•		•	•	•	•	•	•	•
Cowan (38)		•	•	•		•	•	•		•		•
Young (27)	•	•	•	•	•	•	•					•
Cowan (29)	•	•	•	•	•	•	•					•
McIntosh et al. (32)	•	•	•	•	•	•	•					•
Salm Ward et al. (33)	•	•			•	•	•				•	•
Kemp et al. (36)		•		•	•	•	•	•				•
Ahlers-Schmidt et al. (41)	•	•	•	•		•					•	•
Ahlers-Schmidt et al. (40)	•	•		•		•					•	•
Hutton et al. (35)		•		•	•	•	•					•
Hauck et al. (30)	•	•	•	•	•							•
Dillon (34)		•			•	•	•	•				•
Burd et al. (43)		•	•		•	•	•					•
Olds et al. (37)		•			•	•		•				•
Ostfeld et al. (42)		•	•			•					•	•
Mathews et al. (46)		•		•					•			•
Baddock et al. (25)	•			•								•
Engel et al. (28)	•	•	•	•								•
Rienks and Oliva (44)		•	•									•
Carlins et al. (26)	•											•
Yuill et al. (31)	•	•	•	•	•							
Moon et al. (47)		•							•			
Carlin et al. (45)		•							•			

## Discussion

There is good evidence that multi-modal interventions that provide a safe infant sleep space for use both in and out of the parental bed, along with comprehensive face to face safer sleep education programs are effective, delivering improvement across several key outcome measures for safer sleep and safe baby practices in vulnerable families. Safe sleep space (equipment) provision was assessed in combination with other elements, however, most studies reported high percentage of parental use of the safe sleep space provided, even where knowledge scores varied. Therefore, consideration of equipment provision alongside current health and social care provision in the UK may be a useful approach to consider as basic provision when resources are stretched. This has been seen in the proliferation of cardboard baby box schemes in the England since 2016 (49). However, the adoption of these programs is not without criticism and a number of concerns, including infant safety, have been identified. While there is no evidence to support that using a cardboard box for infant sleep reduces the risk for SIDS, some of these schemes are being marketed on this basis. Of more concern is that some of these schemes are being provided through commercial partnerships with health and social care services, which parents are likely to view as an endorsement to the safety of these products. The cardboard baby box schemes were not included in the systematic review as they were widely distributed and outcome data specific to high-risk groups was not available. However, 86% of parents reported that they intended to use the cardboard box for infant sleep, which supports the notion that parents are receptive to accepting an infant sleep space provided to them, data supported by Yuill's (31) feasibility study to introduce the Pepi-Pod program into the UK, which offers an evidence-based and safer alternative to the cardboard box. Several interventions engage peer educators or a mechanism of “paying-it-forward,” using intervention participants to spread infant safety messages further into communities and those traditionally viewed “hard to reach” and vulnerable populations. Such interventions offer a scalable and achievable method to share safer sleep messages which need not be resource heavy. However, some concerns identified with these approaches are the potential for loss of control of fidelity of the messages being communicated by link or peer educators, and the potential that relevant and culturally appropriate peer supporters can be challenging to engage and/or retain. Targeted and long-term evidence-based interventions with continuity of service provider, delivered in the context of enabling parent-provider relationships has benefits for infants and families. The initial contact can be built upon to provide support for parents and opportunities for professionals to identify changes in both the sleep environment and infant care practices, which might decrease the risk of SUDI and SIDS as the infant grows and develops, and family circumstances change. Interventions that have been subsumed into “usual service provision” have delivered sustainable improvements in reducing risks for SUDI and SIDS for infants, and resultant decreases in infant mortality rates. One digital intervention was available for review (45) and was not identified as effective in reporting knowledge improvement and behavior change, except for reducing the use of soft bedding. However, digital interventions are potentially scalable and low cost, and are becoming more popular, particularly with the current SARS-COV-2 pandemic driving the need to find alternative delivery options. It might also be argued that this generation, and future generations of parents are more tech savvy than previous generations, providing an opportunity to capitalize on digital intervention options, and future research should consider approaches to improve the effectiveness and relevance of digital health interventions for families with children considered to be at increased risk of SUDI. One media campaign was reviewed (44), and while no improvements in knowledge were observed, it was identified that targeted campaigns may be successful in raising awareness in the population of interest. This was demonstrated in the national “Back to Sleep” campaign of the early 1990's, which had significant impact on the infant mortality rate at the time. Since then, there have been small localized safe sleep campaigns, but perhaps consideration of another national safer sleep campaign might be useful in raising awareness to a new generation of parents and coupled with targeted interventions that are considered relevant by the population of interest, could offer a cohesive approach to SUDI risk reduction.

While much of the data reported on in these intervention papers were parental self-report, and reported parental behavioral intention, several studies identify decreases in infant mortality and SIDS rates, which, while not shown to be a clear consequence of the interventions, raise the possibility that increased knowledge and adherence to safer sleep recommendations is a valid outcome of these interventions. In considering the evidence to support the development of new interventions, research would be required to understand the relevance and appropriateness for delivery to the UK target population. Seven of the 23 intervention papers used ethnicity as a marker of risk for SUDI, these studies are relevant where characteristics or behavior that increases risk for SUDI in the UK population are described. While parental motivations for certain behaviors may be culturally different, the principal of exhibiting that behavior increasing risk for SUDI should be explored when considering potential application to the UK setting. Interventions also need to have a sound theoretical foundation, for example the Health Belief model (50, 51) or the behavior change wheel (COM-B model) (52). Behavioral models support the assumptions about the links between the intervention and behavior change outcomes and should be clearly stated. To support this, interventions should have clear explanations, considering the needs for parents/carers to be provided with credible advice that incorporates mechanisms of protection which are understandable, and account for the changing needs of a sleeping infant. Intervention design should be collaborative between parents and professionals and consider incorporating robust evaluation and methods of measuring actual practice rather than parental knowledge and intention.

The strengths of this systematic review were that searches of the gray literature and a snowballing approach of relevant citations within the references of the selected records produced a further 42 papers in addition to the 3,506 records identified by the initial database searches; this suggests that our search terms were comprehensive. The agreement rate between authors on selection of included papers was high, and enough papers were identified for meaningful discussion. There are several limitations to this work. The quality of the intervention papers reviewed is variable and synthesis is difficult given the disparate ways in which studies have been reported. While eight were RCT's using large samples and reporting robust results, the remainder of papers reported evaluations or mixed methods approaches potentially impacting on the quality and robustness of reported evidence. The lack of controlled observations in some studies or comparing intentions of infant care practice to actual practice is often very different and leads to a weak design and questionable conclusions. To include papers on interventions specific to high-risk populations, we relied on individual studies' definitions of “high-risk,” meaning that included studies relate to a variety of populations which was necessary as “high-risk” populations vary across cultures and countries. While this means that our conclusions are drawn from a wider pool of literature, it does mean that care must be taken to consider the specific circumstances of, and relevance to, UK high-risk families. We restricted included studies to those which were targeted to higher risk groups, and while the justification for this is clear, it does also mean that we did not include interventions for the general population (e.g., Cardboard baby box schemes) as we would not be able to review their impact in high-risk families separately.

## Conclusion

This paper reports the findings from one arm of a wider systematic review to identify current evidence about how best to increase uptake of safer sleep advice in families with infants considered to be at risk of harm through abuse or neglect. Overall, we found evidence suggestive of how future interventions might be designed to achieve a large scale, targeted approach to risk reduction in families where the infants are considered to be most at risk of SUDI. Interventions should, ideally, be delivered face to face, and from the evidence, innovations that consider how to capitalize on leverage from peer-to-peer models may be of use in this context. Parents and carers require evidence-based advice so they can make decisions on how to keep their infants safe and health professionals should be provided with consistent advice that can be delivered using plain language to families, with plausible explanations as to why this advice will keep their infant safe. Advice should consider parents' own experience and tailor the content of safer sleep conversations to individual families' needs, while also taking account of how to include partners, peers, and wider family members, to extend knowledge and understanding of safer sleep and safe infant care practices to all those who may be caring for a young baby. Further research into how to translate successful interventions for appropriate and relevant application to the UK target population is required. Intervention design should be collaborative between parents and professionals and must include robust evaluation and methods of measuring infant care practice rather than parental knowledge and behavior intention.

## Data Availability Statement

The datasets presented in this study can be found in online repositories. The names of the repository/repositories and accession number(s) can be found below: NCBI with the accession number PRJNA778186 (https://www.ncbi.nlm.nih.gov/sra?linkname=bioproject_sra_all&from_uid=778186).

## Author Contributions

AP, PB, PF, CE, JG, and DW led the review, designed the scope of the work, and wrote the protocol. AP conducted the searches with support on terms from PF, PB, CE, JG, and DW. CE, JG, AP, and DW screened the titles, abstracts and full texts, and discussed final papers for inclusion. Themes were discussed between all authors *via* input into drafts of the final report. All authors contributed to the writing of the manuscript drafts providing comments and changes until a final manuscript for submission was agreed.

## Funding

This work was commissioned by the Child Safeguarding Panel Funding Ref No. RDx135, as part of its review into sudden unexpected death in infancy.

## Conflict of Interest

The authors declare that the research was conducted in the absence of any commercial or financial relationships that could be construed as a potential conflict of interest.

## Publisher's Note

All claims expressed in this article are solely those of the authors and do not necessarily represent those of their affiliated organizations, or those of the publisher, the editors and the reviewers. Any product that may be evaluated in this article, or claim that may be made by its manufacturer, is not guaranteed or endorsed by the publisher.
