# Supplementary material for: Interventions to Improve Safer Sleep Practices in Families With Children Considered to Be at Increased Risk for Sudden Unexpected Death in Infancy: A Systematic Review
Source: Front Pediatr. 2022 Jan 3;9:778186. doi: 10.3389/fped.2021.778186 (PMC8762353; doi:10.3389/fped.2021.778186)
Supplement: Supplementary file 1 [file Data_Sheet_1.docx]

| **Aspect** | **Keywords/ free text** | **Example controlled vocab terms (MeSH, Emtree, CINAHL Headings, PsycINFO, Thesaurus)** |
| --- | --- | --- |
| SUDI terms | Sudden Infant Death*.mp | Exp Sudden Infant Death/ |
|  | SIDS.mp |  |
|  | SUDI.mp |  |
|  | SUID.mp |  |
|  | ASSB.mp |  |
|  | Accidental Suffocation and Strangulation in Bed.mp |  |
|  | (Asphyxia not birth asphyxia not perinatal asphyxia).mp | Asphyxia/ |
|  | (Unexpected death* not SUDEP not epilepsy).mp |  |
|  | Sleep-related death*.mp |  |
|  | Crib death*.mp |  |
|  | Cot death*.mp |  |
|  | Unexplained infant death*.mp |  |
| High-risk groups | Child abuse.mp |  |
|  | High risk*.mp |  |
|  | Vulnerab*.mp |  |
|  | Socioeconomic factor*.mp | Exp Socioeconomic factors/ |
|  | Adverse Childhood Experience*.mp | Exp Adverse Childhood Experiences/ |
|  | Social Marginali#ation*.mp | Social Marginalization/ |
|  | Child neglect*.mp |  |
|  | Child maltreatment*.mp |  |
|  | Substance-related disorder*.mp | Substance-related disorders/ |
|  | Preventive Health Service*.mp | Preventive Health Services/ |
|  | Parenting.mp | Parenting/ |
|  | Maternal deprivation.mp | Maternal deprivation |
| Intervention | Intervention*.mp |  |
|  | Risk reduction*.mp |  |
|  | Injury prevention*.mp | Accident prevention/ |
|  | Health education*.mp | Exp Health education/ |
|  | Health behavio?r*.mp | Exp Health behavior/ |
|  | Education*.mp |  |
|  | Infant equipment*.mp | Exp Infant equipment/ |
|  | Printed education material*.mp |  |
|  | Maternal behavio?r*.mp | Exp Maternal behavior/ |
|  | Parent* education*.mp | Caregivers/ed |

Appendix 1: Example search with key terms
